# Ultrasound-Guided Injection of the Posterior Talofibular Ligament Using a Bony Landmark-Based Approach: A Technical Report With Cadaveric Demonstration of Feasibility

**DOI:** 10.7759/cureus.112703

**Published:** 2026-07-15

**Authors:** Yonghyun Yoon, Sang-Hyun Kim, U-Young Lee, Jihyo Hwang, Teinny Suryadi, King Hei Stanley Lam

**Affiliations:** 1 Orthopaedics, International Academy of Musculoskeletal Medicine, Hong Kong, HKG; 2 Orthopaedics, International Academy of Regenerative Medicine, Incheon, KOR; 3 Orthopaedics, MSKUS, San Diego, USA; 4 Orthopaedic Surgery, Hallym University Kangnam Sacred Heart Hospital, Seoul, KOR; 5 Orthopaedic Surgery, Incheon Terminal Orthopedic Surgery Clinic, Incheon, KOR; 6 Anatomy, College of Korean Medicine, Woosuk University, Jeonbuk-do, KOR; 7 Anatomy, Catholic Institute for Applied Anatomy, College of Medicine, Catholic University of Korea, Seoul, KOR; 8 Physical Medicine and Rehabilitation, Medistra Hospital, Jakarta Pusat, IDN; 9 Physical Medicine and Rehabilitation, Synergy Clinic, Jakarta, IDN; 10 Physical Medicine and Rehabilitation, Hermina Podomoro Hospital, Jakarta, IDN; 11 Clinical Research, International Academy of Regenerative Medicine, Incheon, KOR; 12 Clinical Research, The Indonesian Malaysian Regenerative Institute, Jakarta, IDN; 13 Clinical Research, The International Association of Musculoskeletal Medicine, Kowloon, HKG; 14 Faculty of Medicine, The Chinese University of Hong Kong, New Territories, HKG; 15 Faculty of Medicine, The University of Hong Kong, Hong Kong, HKG; 16 Clinical Research, The Hong Kong Institute of Musculoskeletal Medicine, Kowloon, HKG

**Keywords:** ankle instability, bony landmark, cadaveric feasibility, flexor hallucis longus, musculoskeletal ultrasound, posterior talofibular ligament, ptfl, regenerative medicine, technical report, ultrasound-guided injection

## Abstract

The posterior talofibular ligament (PTFL) is the deepest and strongest component of the lateral ankle ligament complex, yet it is infrequently evaluated or targeted in routine musculoskeletal ultrasound practice because of its deep location and surrounding anatomy. This technical report describes a structured ultrasound-guided PTFL injection technique based on sequential bony landmark identification and dynamic localization of the flexor hallucis longus (FHL) tendon. Ultrasound images demonstrating the scanning protocol were obtained from a healthy volunteer after written informed consent. A cadaveric demonstration of feasibility was then performed in one fresh-frozen specimen using ultrasound-guided injection of 2 mL of a commercially available cosmetic hyaluronic acid-based filler mixed with green dye at the talar insertional region of the PTFL, followed by layer-by-layer dissection. The scanning approach allowed identification of the PTFL through cranial-to-caudal assessment of the posterior ankle, medial probe translation to define the posterior malleolus and posterior process of the talus, and dynamic confirmation of the FHL tendon. Gross dissection demonstrated focal dye localization in the region of the PTFL talar insertion, predominantly beneath the transverse intermuscular septum, without gross spread to adjacent superficial neurovascular structures in this specimen. These findings support the anatomical plausibility of the intended target region in this technical setting. In a potential clinical context, PTFL-region injection may be relevant in selected cases of persistent posterior ankle symptoms, posterior impingement, or incompletely explained instability, either for diagnostic symptom localization or as a framework for future targeted therapeutic interventions. However, because feasibility assessment was limited to a single cadaveric specimen and gross dissection alone, and because the surrogate cadaveric injectate was selected for anatomical visualization rather than treatment simulation, the technique should not be interpreted as establishing selective intraligamentous placement, optimal therapeutic injectate volume, clinical efficacy, procedural accuracy, or formal safety. Further anatomical, prospective, and comparative studies are warranted.

## Introduction

The lateral ligament complex of the ankle is composed of three primary structures: the anterior talofibular ligament (ATFL), calcaneofibular ligament (CFL), and posterior talofibular ligament (PTFL) [1]. Among these, the ATFL is the most commonly injured ligament and has therefore been extensively studied in the setting of ankle sprains and chronic lateral ankle instability [2]. In contrast, the PTFL has received comparatively limited attention in both clinical and imaging-based research despite being the deepest and strongest component of the lateral ligament complex [3].

In commonly used injury classification systems, such as the Lauge-Hansen classification, emphasis is placed primarily on syndesmotic structures including the anterior inferior tibiofibular ligament and posterior inferior tibiofibular ligament (PITFL) [4]. The PTFL is often overlooked, although it contributes to posterior ankle stability and may be relevant in persistent posterior ankle pain, impingement, or residual instability [3].

Anatomically, the PTFL originates from the malleolar fossa on the medial aspect of the lateral malleolus and inserts onto the lateral tubercle of the posterior process of the talus [5]. Despite its distinct anatomical course, it is not routinely visualized or targeted during conventional ultrasound examinations because of its deep location and the presence of overlying structures [6,7].

The flexor hallucis longus (FHL) tendon passes between the medial and lateral tubercles of the posterior process of the talus and can serve as a reliable dynamic sonographic landmark [8]. Once the FHL tendon is identified, the posterior process of the talus can be localized more confidently, thereby facilitating visualization of the talar insertion of the PTFL.

Although prior studies have described the anatomy and imaging appearance of the PTFL, a standardized and clinically applicable ultrasound-guided interventional approach remains limited [9,10]. A structured approach to this region may be relevant in selected cases of persistent posterior ankle symptoms, posterior impingement, or incompletely explained instability, particularly when PTFL-region pathology is suspected and more superficial lateral ligament findings do not fully account for symptoms. In principle, such an approach could be used either diagnostically, for example, with a small-volume local anesthetic injection to help localize symptoms, or as a framework for future targeted therapeutic or regenerative interventions. However, the present study did not evaluate clinical outcomes, therapeutic injectate selection, or optimal treatment volume. The purpose of this technical report is therefore to describe a structured ultrasound-guided PTFL injection technique using a bony landmark-based scanning protocol with dynamic FHL identification and to provide cadaveric evidence supporting the anatomical feasibility of the intended injectate location.

## Technical report

Ethical considerations

This cadaveric study was reviewed by the Institutional Review Board of the Catholic University of Korea and was granted exemption from full ethical review because it involved cadaveric specimens only and did not include identifiable personal data (IRB No. MIRB-20260120-006). Ultrasound images used to demonstrate the scanning protocol were obtained from a healthy volunteer after written informed consent.

Ultrasound scanning protocol and landmark identification

To demonstrate the ultrasonographic anatomy of the posterior ankle and the PTFL, ultrasound images illustrating the scanning protocol were obtained from a healthy volunteer.

Ultrasonographic evaluation was performed using an Alpinion XC90 Elite ultrasound system (ALPINION Medical Systems Co., Ltd., Seoul, Republic of Korea) equipped with a linear transducer. Imaging parameters were standardized with a depth setting of 4 cm and a dynamic range of 60 dB.

The subject was positioned prone with the ankle maintained in slight plantarflexion. A systematic bony landmark-based scanning approach was used to identify the PTFL.

Step 1: Cranial-to-caudal posterior ankle scanning

**Figure 1:**
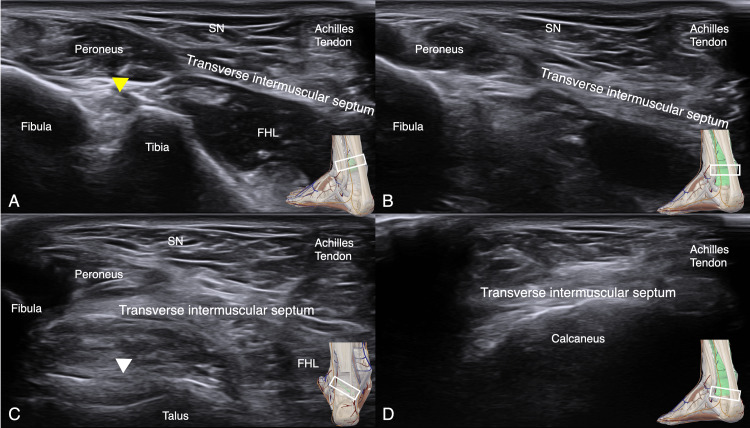
Sequential ultrasound scanning of the posterior ankle in the prone position from cranial-to-caudal levels. The transducer was moved sequentially from superior to inferior using pivoting and short-axis linear sliding to evaluate the anatomical structures at each level. The corresponding anatomy at each scanning level is illustrated in the inset schematic diagrams, with the white rectangle indicating the transducer position. (A) Posterior inferior tibiofibular ligament (PITFL). (B) Ankle joint. (C) Posterior talofibular ligament (PTFL). (D) Calcaneus. Yellow triangle: PITFL. White triangle: PTFL. Image courtesy of Dr. Yonghyun Yoon and Prof. King Hei Stanley Lam. SN: sural nerve; PITFL: posterior inferior tibiofibular ligament; PTFL: posterior talofibular ligament.

The transducer was initially positioned at the inferior tip of the lateral malleolus in the axial plane. Sequential cranial-to-caudal scanning was then performed to identify the PITFL, the ankle joint, the PTFL level, and the calcaneus (Figure 1).

Step 2: Medial translation to define osseous landmarks

**Figure 2:**
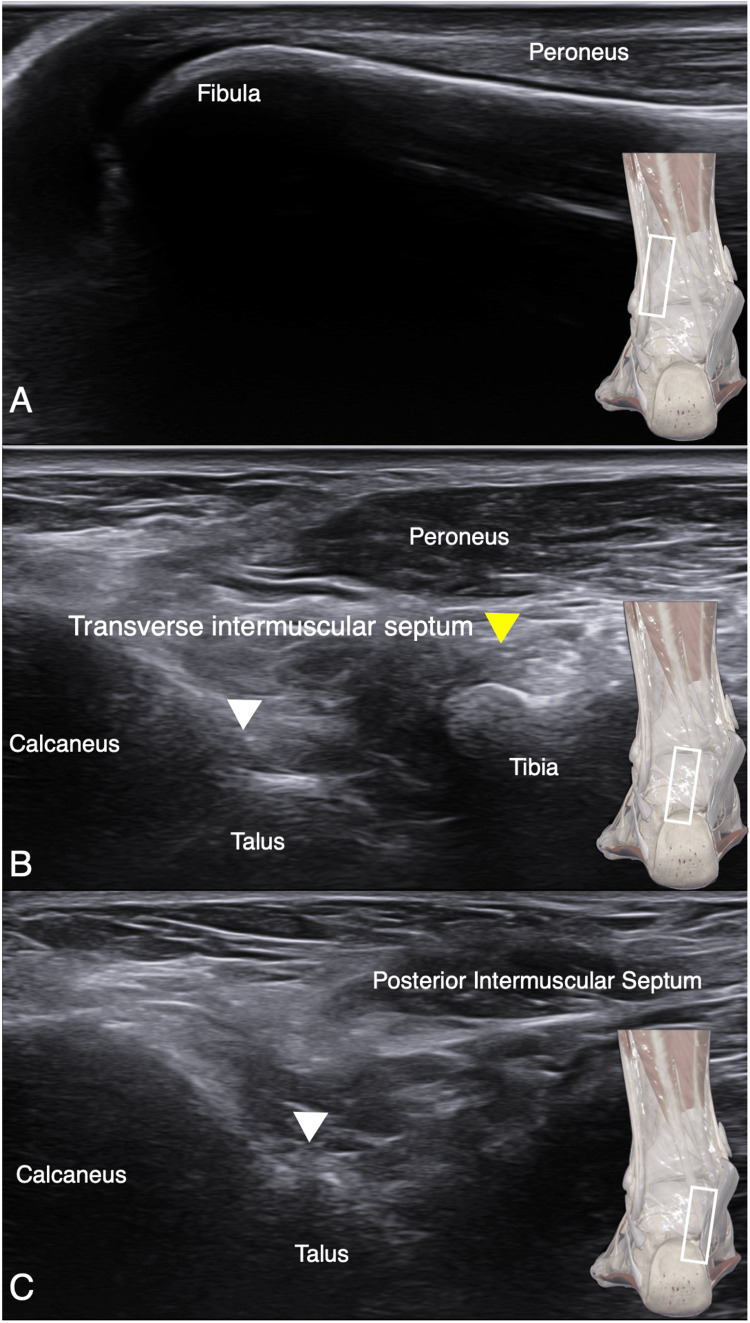
Sagittal scanning of the posterior ankle from the fibula toward the tibia in the prone position. The transducer was translated from lateral to medial to delineate the anatomical transition from fibular to tibial structures. (A) Fibular level. (B) PITFL attachment over the posterior malleolus of the tibia. (C) Posterior process of the talus with PTFL attachment. Yellow triangle: PITFL. White triangle: PTFL. Image Courtesy of Dr. Yonghyun Yoon and Prof. King Hei Stanley Lam. PITFL: posterior inferior tibiofibular ligament; PTFL: posterior talofibular ligament.

The transducer was translated medially from the fibula toward the tibia to define key osseous landmarks, especially the posterior malleolus and the posterior process of the talus (Figure 2).

Step 3: Dynamic identification of the FHL tendon

**Figure 3:**
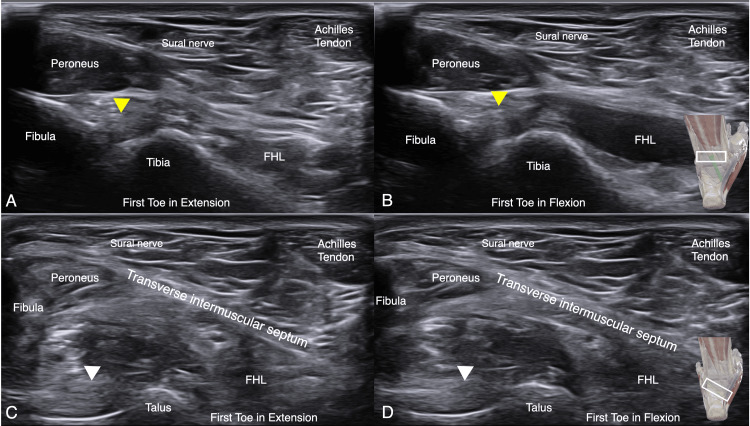
Identification of the posterior talofibular ligament (PTFL) using the flexor hallucis longus (FHL) tendon as a dynamic landmark. (A and B) Dynamic identification of the FHL tendon at the PITFL level during great toe flexion and extension. (C and D) Visualization of the PTFL attachment lateral to the FHL tendon at the level of the posterior process of the talus. Yellow triangle: PITFL. White triangle: PTFL. Image courtesy of Dr. Yonghyun Yoon and Prof. King Hei Stanley Lam. PITFL: posterior inferior tibiofibular ligament; PTFL: posterior talofibular ligament; FHL: flexor hallucis longus.

Dynamic assessment during great toe flexion and extension was performed to identify the FHL tendon, which served as a key landmark for PTFL localization. At the level of the posterior process of the talus, the PTFL was visualized lateral to the FHL tendon (Figure 3).

Cadaver preparation

The injection experiment was performed using one fresh-frozen cadaveric specimen from a 76-year-old female donor. The specimen was positioned prone with the ankle maintained in slight plantarflexion to replicate the clinical injection setting and facilitate access to the posterior ankle structures.

Injection technique

Ultrasound-guided injection was performed using a 23-gauge, 6-cm needle advanced in a cranial-to-caudal direction under real-time ultrasound guidance with an out-of-plane approach.

The intended target was the talar insertional region of the PTFL. This target region was defined sonographically using the sequential scanning protocol described above: (1) cranial-to-caudal posterior ankle scanning to identify the PITFL, ankle joint, PTFL level, and calcaneus; (2) medial transducer translation to identify the posterior malleolus and posterior process of the talus; and (3) dynamic identification of the FHL tendon during great toe flexion and extension. The needle was advanced toward the region lateral to the FHL tendon at the level of the posterior process of the talus.

An out-of-plane approach was selected because the posterior talar target region presents a relatively narrow sonographic corridor, making short-axis target centering practical in this technical setting. In addition, this approach may allow access to different portions of the PTFL by adjusting the needle direction after initial placement. However, this choice should be interpreted as a technical preference rather than comparative evidence. An in-plane approach may offer more continuous needle visualization, but in some ankles, it may be technically constrained by the limited acoustic window and the oblique trajectory required to approach the target region.

**Figure 4:**
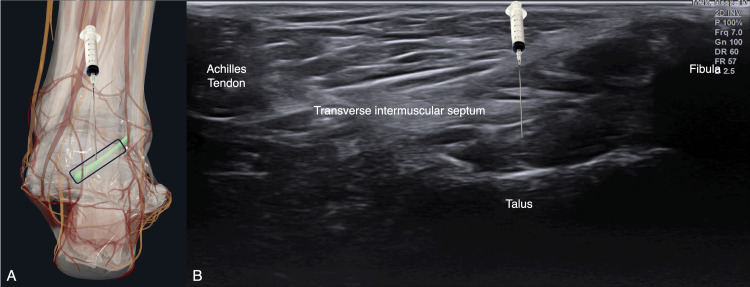
Ultrasound-guided PTFL injection using an out-of-plane approach. (A) Schematic illustration of the intended needle trajectory toward the talar insertion of the PTFL. (B) Real-time ultrasound-guided injection demonstrating needle placement at the target region. The cadaveric injectate consisted of 2 mL of a commercially available cosmetic hyaluronic acid-based filler mixed with green dye for gross anatomical assessment of injectate distribution. Image courtesy of Dr. Yonghyun Yoon and Prof. King Hei Stanley Lam. PTFL: posterior talofibular ligament.

Because selective intraligamentous placement could not be confirmed sonographically, the procedural goal was placement at the intended PTFL talar insertional region rather than proof of intraligamentous delivery. A total of 2 mL of surrogate injectate was then delivered for cadaveric assessment. The surrogate injectate consisted of a commercially available cosmetic hyaluronic acid-based filler mixed with green dye to facilitate focal gross identification during subsequent dissection. A relatively high-viscosity material was selected to reduce unintended spread beyond the intended footprint and to facilitate visual localization at dissection. This cadaveric injectate was used as an anatomical tracing surrogate and was not intended to represent an optimal therapeutic injectate or clinical treatment volume (Figure 4).

Cadaveric dissection procedure

Following ultrasound-guided injection, anatomical assessment was performed through meticulous layer-by-layer cadaveric dissection.

After removal of the skin and subcutaneous tissue, the transverse intermuscular septum and adjacent superficial neurovascular structures, including the sural nerve and fibular artery, were identified. The septum was then carefully reflected to expose the deep posterior ankle compartment for evaluation of dye distribution.

Cadaveric feasibility findings

**Figure 5:**
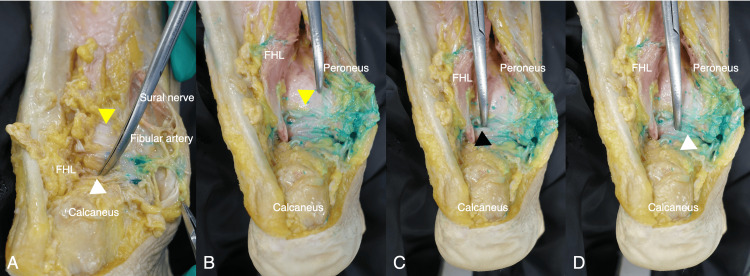
Cadaveric demonstration of injectate distribution following PTFL injection. (A) Superficial dissection layer showing the transverse intermuscular septum and adjacent neurovascular structures. (B) Deep dissection layer after reflection of the transverse intermuscular septum. (C) Posterior intermalleolar ligament, also known as the transverse tibiofibular ligament. (D) PTFL. Yellow triangle: PITFL attachment site. White triangle: PTFL attachment site. Black triangle: posterior intermalleolar ligament attachment site. Image courtesy of Dr. Yonghyun Yoon and Prof. King Hei Stanley Lam. PTFL: posterior talofibular ligament; PITFL: posterior inferior tibiofibular ligament; FHL: flexor hallucis longus.

Gross dissection demonstrated that the surrogate injectate was predominantly localized within the deep posterior ankle compartment beneath the transverse intermuscular septum. Green dye was observed in the region of the PTFL talar insertion, in proximity to the posterior process of the talus and adjacent to the expected PTFL course, supporting the anatomical plausibility of the intended target region in this specimen (Figure 5). For the purpose of this feasibility report, target-region localization was interpreted on the basis of focal gross dye presence at the intended talar insertional region relative to the PTFL, posterior talar process, and neighboring FHL landmark. However, these gross findings do not confirm selective intraligamentous placement.

No gross dye spread to adjacent superficial neurovascular structures was observed in this specimen. Likewise, the planned needle trajectory appeared grossly separate from the identified superficial neurovascular structures during dissection. These observations should be interpreted as specimen-specific findings only and not as evidence of formal procedural safety.

The high-viscosity nature of the surrogate injectate may have favored focal retention and limited spread, thereby influencing the observed localization pattern. Accordingly, the cadaveric spread pattern should not be assumed to predict the behavior of lower-viscosity therapeutic injectates in vivo.

Technical pearls and procedural considerations

Several practical observations emerged during iterative refinement of this technique.

First, sequential use of bony landmarks improved orientation in this anatomically deep region. Beginning at the lateral malleolus and then progressing to the posterior malleolus and posterior process of the talus provided a structured framework for localization.

Second, the FHL tendon was a particularly useful dynamic landmark. Great toe flexion and extension allowed real-time confirmation of tendon location and facilitated identification of the PTFL region lateral to the tendon.

Third, approaching the talar insertional region of the PTFL provided a practical sonographic working corridor in the prone position during technique development. This observation should be regarded as a procedural impression rather than comparative evidence demonstrating superiority over alternative target regions such as the fibular origin.

Fourth, the out-of-plane approach used in this report was selected as a practical means of maintaining target-region centering within a constrained posterior ankle window. This should not be interpreted as evidence that out-of-plane guidance is superior to in-plane guidance. In-plane guidance may provide more continuous needle visualization but may be technically more difficult at some angles depending on local anatomy and access angle.

Fifth, real-time ultrasound guidance remains essential. Although no gross spread to adjacent superficial neurovascular structures was observed in this single cadaveric specimen, the present report does not constitute a formal safety study, and these findings should be interpreted accordingly.

## Discussion

This technical report describes a structured ultrasound-guided approach for identifying and targeting the PTFL using sequential bony landmarks and dynamic FHL localization. Its principal contribution is procedural rather than inferential, as it presents a practical sonographic workflow for a ligament that is rarely assessed directly in routine musculoskeletal ultrasound practice.

Current evaluation and treatment of ankle instability generally focus on the ATFL and, to a lesser extent, the CFL [11,12]. While this emphasis is understandable given the frequency of ATFL injury, posterior stabilizing structures may be under-recognized in patients with persistent posterior ankle symptoms or incompletely explained instability [3]. Because the PTFL lies deep within the posterior ankle and is less accessible than the more superficial lateral ligaments, it has not been routinely incorporated into ultrasound-guided interventional practice [6,7,9,10].

A strength of the present technique is its reliance on structured anatomical orientation. Sequential identification of the PITFL level, ankle joint, posterior talar process, and FHL tendon provides a systematic method for approaching the PTFL despite its deep position. The use of a dynamic landmark is particularly helpful because it supplements static bony mapping with functional confirmation.

Cadaveric dissection supported the anatomical plausibility of the described technique by demonstrating dye deposition in the region of the PTFL talar insertion in this specimen. However, these findings should be interpreted cautiously. This was a single-cadaver feasibility study, and the assessment of injectate distribution was gross rather than histologic. Accordingly, the present report does not establish selective intraligamentous placement, reproducibility across operators, clinical efficacy, procedural accuracy in clinical practice, or formal procedural safety.

Anatomical variability may also influence the applicability of this technique. Variation in the morphology of the posterior process of the talus, the presence or configuration of an os trigonum, and the dual-bundle or variable insertional anatomy of the PTFL may alter the sonographic appearance of the posterior ankle and the accessibility of the intended target region [5,8]. Likewise, the relative position of the FHL tendon and surrounding posterior ankle structures may vary across individuals. These factors may affect visualization, needle trajectory selection, and the ease with which the described workflow can be applied.

The cadaveric injectate used in this study was selected for anatomical visualization rather than treatment simulation. Specifically, a high-viscosity cosmetic hyaluronic acid-based filler mixed with green dye was used to facilitate focal gross identification at dissection and to reduce unintended dispersal. While this was useful for anatomical feasibility assessment, it may also have favored focal retention and limited spread. Therefore, the observed dye distribution should not be interpreted as defining an optimal therapeutic injectate, expected in vivo spread characteristics, or an appropriate clinical treatment volume.

From a clinical perspective, the present report may offer an educational framework for image-guided evaluation of posterior ankle anatomy and may help inform the design of future interventional studies targeting this region. Potential purposes of PTFL-region injection may include diagnostic symptom localization in selected cases of persistent posterior ankle pain, posterior impingement, or incompletely explained instability, as well as exploration of future targeted therapeutic strategies. In a diagnostic setting, a small-volume local anesthetic injection might be considered to minimize non-specific spread. In a therapeutic or regenerative setting, a small-volume periligamentous injectate might also be considered, but the optimal injectate type and volume for the PTFL region remain unknown. Although regenerative injection strategies such as prolotherapy, dextrose-based injections, and platelet-rich plasma have been described in broader musculoskeletal practice [13-16], the present study did not evaluate treatment effect, injectate performance, or patient outcomes. Therefore, no conclusions regarding therapeutic benefit, optimal clinical injectate, or appropriate treatment volume can be inferred from this cadaveric feasibility study, and any such applications remain speculative.

Limitations

This report has several limitations. First, a cadaveric feasibility assessment was performed on only one specimen. Second, the ultrasonographic demonstration was obtained from a single healthy volunteer. Third, evaluation relied on gross dissection without histologic confirmation, and therefore, selective intraligamentous placement could not be established. Fourth, cadaveric injectate spread may not reflect in vivo behavior. In particular, the high-viscosity surrogate injectate used in this study may have favored focal retention and limited spread and therefore should not be assumed to represent the behavior of clinically used therapeutic injectates. Fifth, no inter-rater reliability, clinical outcome assessment, or formal safety analysis was performed. Sixth, the findings may not fully account for anatomical variability of the posterior ankle, including variation in PTFL morphology, posterior talar process anatomy, os trigonum configuration, and the local relationship of the FHL tendon to adjacent structures. Finally, no comparison was made with alternative ultrasound-guided or non-ultrasound-guided targeting techniques.

## Conclusions

This technical report describes a structured ultrasound-guided technique for approaching the PTFL talar insertional region using a bony landmark-based protocol and dynamic identification of the FHL tendon. Cadaveric findings from a single specimen support the anatomical plausibility of the described approach and indicate that the intended target region was approachable under ultrasound guidance in this technical setting. This technique may serve as an anatomical and procedural framework for future diagnostic or targeted therapeutic investigation of the PTFL region in selected posterior ankle conditions. However, because the feasibility assessment was limited to one cadaveric specimen and gross dissection alone, and because the surrogate injectate was selected for anatomical visualization rather than treatment simulation, the present findings should not be interpreted as establishing selective intraligamentous placement, optimal therapeutic injectate volume, procedural safety, reproducibility, procedural accuracy in clinical practice, or clinical effectiveness. Further, anatomical, technical, and prospective clinical studies are needed.
